# Antibody-Mediated Inhibition of CTLA4 Aggravates Atherosclerotic Plaque Inflammation and Progression in Hyperlipidemic Mice

**DOI:** 10.3390/cells9091987

**Published:** 2020-08-29

**Authors:** Kikkie Poels, Mandy M. T. van Leent, Myrthe E. Reiche, Pascal J. H. Kusters, Stephan Huveneers, Menno P. J. de Winther, Willem J. M. Mulder, Esther Lutgens, Tom T. P. Seijkens

**Affiliations:** 1 Department of Medical Biochemistry, Amsterdam Cardiovascular Sciences (ACS), Amsterdam University Medical Centers, University of Amsterdam, 1105AZ Amsterdam, The Netherlands; k.poels@amsterdamumc.nl (K.P.); mandy.vanleent@mountsinai.org (M.M.T.v.L.); m.e.reiche@amsterdamumc.nl (M.E.R.); p.j.kusters@amsterdamumc.nl (P.J.H.K.); s.huveneers@amsterdamumc.nl (S.H.); m.dewinther@amsterdamumc.nl (M.P.J.d.W.); willem.mulder@mountsinai.org (W.J.M.M.); e.lutgens@amsterdamumc.nl (E.L.); 2 Biomedical Engineering and Imaging Institute, Icahn School of Medicine at Mount Sinai, New York, NY 10029, USA; 3 Laboratory of Chemical Biology, Department of Biomedical Engineering and Institute for Complex Molecular Systems, Eindhoven University of Technology, 5612AZ Eindhoven, The Netherlands; 4 Institute for Cardiovascular Prevention (IPEK), Ludwig Maximilian’s University, 80336 Munich, Germany; 5 German Centre for Cardiovascular Research (DZHK), Partner Site Munich Heart Alliance, 80802 Munich, Germany; 6 Department of Internal Medicine, Amsterdam University Medical Centers, Vrije Universiteit Amsterdam, 1081AV Amsterdam, The Netherlands; 7 Department of Hematology, Amsterdam University Medical Centers, Vrije Universiteit Amsterdam, 1081AV Amsterdam, The Netherlands

**Keywords:** atherosclerosis, Cytotoxic T-Lymphocyte Associated protein (CTLA) 4, T cells, inflammation, immune checkpoint inhibitors

## Abstract

T cell-driven inflammation plays a critical role in the initiation and progression of atherosclerosis. The co-inhibitory protein Cytotoxic T-Lymphocyte Associated protein (CTLA) 4 is an important negative regulator of T cell activation. Here, we studied the effects of the antibody-mediated inhibition of CTLA4 on experimental atherosclerosis by treating 6–8-week-old Ldlr^−/−^ mice, fed a 0.15% cholesterol diet for six weeks, biweekly with 200 μg of CTLA4 antibodies or isotype control for six weeks. ^18^F-fluorodeoxyglucose Positron Emission Tomography—Computed Tomography showed no effect of the CTLA4 inhibition of activity in the aorta, spleen, and bone marrow, indicating that monocyte/macrophage-driven inflammation was unaffected. Correspondingly, flow cytometry demonstrated that the antibody-mediated inhibition of CTLA4 did not affect the monocyte populations in the spleen. αCTLA4 treatment induced an activated T cell profile, characterized by a decrease in naïve CD44^−^CD62L^+^CD4^+^ T cells and an increase in CD44^+^CD62L^−^ CD4^+^ and CD8^+^ T cells in the blood and lymphoid organs. Furthermore, αCTLA4 treatment induced endothelial activation, characterized by increased ICAM1 expression in the aortic endothelium. In the aortic arch, which mainly contained early atherosclerotic lesions at this time point, αCTLA4 treatment induced a 2.0-fold increase in the plaque area. These plaques had a more advanced morphological phenotype and an increased T cell/macrophage ratio, whereas the smooth muscle cell and collagen content decreased. In the aortic root, a site that contained more advanced plaques, αCTLA4 treatment increased the plaque T cell content. The short-term antibody-mediated inhibition of CTLA4 thus accelerated the progression of atherosclerosis by inducing a predominantly T cell-driven inflammation, and resulted in the formation of plaques with larger necrotic cores and less collagen. This indicates that existing therapies that are based on αCTLA4 antibodies may promote CVD development in patients.

## 1. Introduction

Atherosclerosis is a chronic lipid-driven inflammatory disease of the large arteries and a major underlying cause of cardiovascular disease [1,2]. T cell-mediated inflammatory responses drive the development of atherosclerosis. Single-cell RNA sequencing and the mass cytometry of human atherosclerotic plaques recently demonstrated that T cells are a dominant immune cell type in atherosclerotic lesions [3]. Activated CD4^+^ and CD8^+^ T cells promote the initiation of atherosclerosis and also drive the progression towards vulnerable plaques that may trigger myocardial infarction or ischemic stroke upon rupture [4,5]. Immune checkpoint proteins have a critical role in the regulation of T cell activation [6]. Co-stimulatory molecules and co-inhibitory molecules are the predominant members of the immune checkpoint protein family and may either promote or hamper T cell activation. In addition to their classical role in the regulation of T cell activation, co-stimulatory and co-inhibitory molecules facilitate the interaction between lymphoid and myeloid immune cells and non-immune cells, such as endothelial cells, thereby orchestrating the secretion of cytokines and chemokines and cellular proliferation and polarization, which shape the inflammatory response that drives atherosclerosis [7].

The CD80/CD86-CD28 and -CTLA4 pathway is one of the most extensively studied immune checkpoints. CD80 (B7.1) and CD86 (B7.2) are co-stimulatory proteins that are mainly expressed on antigen-presenting cells and have an overlapping function. Interactions of CD80/86 with the co-stimulatory protein CD28 promote CD4^+^ and CD8^+^ T cell activation, survival, and effector functions. On the other hand, interactions with the co-inhibitory molecule CTLA4 limit T cell activation and induce regulatory T cell responses, thereby dampening inflammation [8]. Given their central role in the regulation of inflammatory responses, therapeutic strategies to modulate CD80/CD86-CD28 and -CTLA4 interactions have been implemented in the clinic. For example, the CTLA4-Ig fusion protein abatacept, which prevents CD28-induced CD80/86-mediated immune cell activation, is used for the treatment of autoimmune diseases, including rheumatoid arthritis [9]. On the other hand, the αCTLA4 antibody ipilimumab is used to boost T cell-driven anti-tumor immunity in patients with various types of cancer, including melanoma and non-small cell lung cancer, which led to unparalleled positive responses in these patients [10,11].

Experimental studies have also identified a role for CTLA4 in atherosclerosis. For example, the genetic deficiency of CD80/86 reduced the development of atherosclerosis in *Ldlr^−/−^* mice by hampering T cell-driven inflammatory responses [12]. In addition, the abatacept-mediated inhibition of CD80/CD86-CD28 interactions reduced atherosclerosis in ApoE3*Leiden mice by preventing the activation of CD4^+^ T cells [13]. Whether the antibody-mediated inhibition of CTLA4 affects atherosclerosis is incompletely understood and nowadays is particularly relevant, as antagonistic CTLA4 antibodies are increasingly used in cancer patients. Therefore, we evaluated the effects of the antibody-mediated inhibition of CTLA4 on experimental atherosclerosis in hyperlipidemic mice.

## 2. Materials and Methods

### 2.1. Animal Experiments

Male Ldlr^−/−^ mice (6–8 weeks old)(*n* = 14) were bred and housed at the local animal facility and were fed a 0.15% cholesterol diet ad libitum. At 12 weeks of age, the mice were treated biweekly with an antagonistic CTLA4 antibody (200 µg/injection; IgG2b, clone: 9D9, BE0164, BioXcell, West Lebanon, NH, USA) or isotype control (200 µg/injection; IgG2b, clone: MPC-11, BE0086, BioXcell) for six weeks. All the animal experiments were approved by the Committee for Animal Welfare of the University of Amsterdam, The Netherlands (AVD1180020171666). For the ^18^F-FDG PET studies, female Apoe^−/−^ mice (*n* = 16) were fed a 0.15% cholesterol diet ad libitum, after which they were treated with αCTLA4 antibody or the control for 4 weeks. The animal care and procedures were based on an approved institutional protocol from Icahn School of Medicine at Mount Sinai.

### 2.2. ^18^F-FDG PET-CT Imaging

Anesthesia was induced using ketamine (100 mg/kg) with xylazine (6 mg/kg) intraperitoneally and maintained with a 1% isoflurane (Baxter Healthcare, Deerfield, IL, USA)/oxygen gas mixture. The animals were injected with 266.7 ± 18.3 μCi ^18^F-FDG via the lateral tail vein. Then, 60 min after injection, the animals were imaged using a Mediso nanoScan PET/CT (Mediso, Budapest, Hungary). A whole-body CT scan was performed (energy 50kVp, current 180 μAs, isotropic voxel size at 0.25 mm), followed by a 30 min PET scan. Reconstruction was performed using the TeraTomo 3D reconstruction algorithm, with attenuation correction using the Mediso Nucline software. The coincidences were filtered with an energy window between 400 and 600 keV. The voxel size was isotropic, with a 0.4 mm width, and the reconstruction was applied for 4 full iterations, 6 subsets per iteration. The results are displayed as the mean standardized uptake value (SUVmean).

### 2.3. Gamma Counting

Immediately after the completion of the PET/CT scan, the mice were euthanized and perfused with 20 mL of PBS. Aortas were dissected from the root until the aortic bifurcation, and gently cleaned from the surrounding fat. Femurs (including bone marrow) and spleens were harvested. Tissues were weighted and the radioactivity was measured using a Wizard2 2480 automatic gamma counter (Perkin Elmer, Waltham, MA, USA). The ^18^F-FDG uptake was expressed as the % of injected dose per gram of tissue (%ID/g).

### 2.4. Flow Cytometry

Spleen and lymph nodes were isolated, and single-cell suspensions were obtained by crushing the tissues through 70 µm filters. The spleen and blood samples were subjected to red blood cell lysis (red blood cell lysis buffer: 8.4 g of NH4Cl and 0.84 g of NaHCO3 per liter of distilled water). Single cell suspensions were stained with fluorescently labeled antibodies (CD45, CD3, CD4, CD8, F4/80, Ly6C, Ly6G, MHCI, MHCII, CD44, CD62L, CD19, CD11C, NK1.1, all from BD Biosciences, San Diego, CA, USA). Nonspecific binding was prevented by the pre-incubation of the cells with a Fc receptor-blocking antibody CD16/32 (Ebioscience, Vienna, Austria). Flow cytometry was performed on a BD Canto II (BD Biosciences), and analysis was performed using the Flowjo software version 10 (Tree star, Ashland, OR, USA).

### 2.5. Histology

At the age of 18 weeks, Ldlr^−/−^ mice were sacrificed, and the arterial tree was perfused with PBS and 1% paraformaldehyde (PFA). The aortic arch and organs were isolated and fixed in 1% and 4% paraformaldehyde, respectively. Longitudinal sections of the aortic arch (4 µm) as well as aortic root sections from the heart (4 µm) were stained with hematoxylin and eosin to be analyzed for plaque extent, phenotype, and necrotic core size, as described [14]. Using the Virmani classification, intimal xanthoma (IX) was defined by a small lesion consisting of foam cells in which no extracellular lipid accumulation can be detected. Pathological intimal thickening (PIT) was defined as a larger lesion that mainly consists of macrophage foam cells but contains small extracellular lipid pools and some matrix depositions; a fibrous cap atheroma (FCA) was defined as an advanced atherosclerotic lesion with a clear fibrous cap and necrotic cores (extracellular lipid accumulation, cholesterol crystals and/or calcification). Immunohistochemistry was performed for CD3 (AbD Serotec, Veenendaal, The Netherlands), CD8 (AbD Serotec), MAC3 (BD Pharmingen, San Diego, CA, USA), α-smooth muscle actin (αSMA) (Sigma, Zwijndrecht, The Netherlands), Ki67 (Abcam, Cambridge, United Kingdom). To detect apoptotic cells, sections were TUNEL-stained using the In Situ Cell Death Detection Kit (Roche, Zwijndrecht, The Netherlands). Collagen was detected by sirius red staining [14]. Morphometric analyses were performed on a Leica DM3000 microscope with a DFC 295 camera and the Adobe Photoshop CS6, Image J, or Las4.0 software (Leica, Amsterdam, The Netherlands).

### 2.6. Confocal Microscopy

The abdominal aorta was isolated, fixed in 4% PFA for 12 min, opened in the longitudinal direction, and stained for ICAM1 (Abcam), VCAM1 (Abcam), or VE-cadherin (Abcam) in combination with DAPI. En face sections were obtained on a Leica SP8 confocal microscope, and all the analyses were performed with Image J [15].

### 2.7. Statistics

Data are depicted as mean ± SD. Data were analyzed by either using an unpaired Student’s *t*-test or by a non-parametric Mann–Whitney ranking test depending on the normality determined by the D’Agostino–Pearson omnibus normality test. For all analyses, the Graphpad Prism 5.0 software (GraphPad Software Inc., San Diego, CA, USA) was used. *p*-values < 0.05 were considered significant.

## 3. Results

### 3.1. Antibody-Mediated Inhibition of CTLA4 Does Not Affect Monocyte/Macrophage-Driven Vascular Inflammation

**Figure 1:**
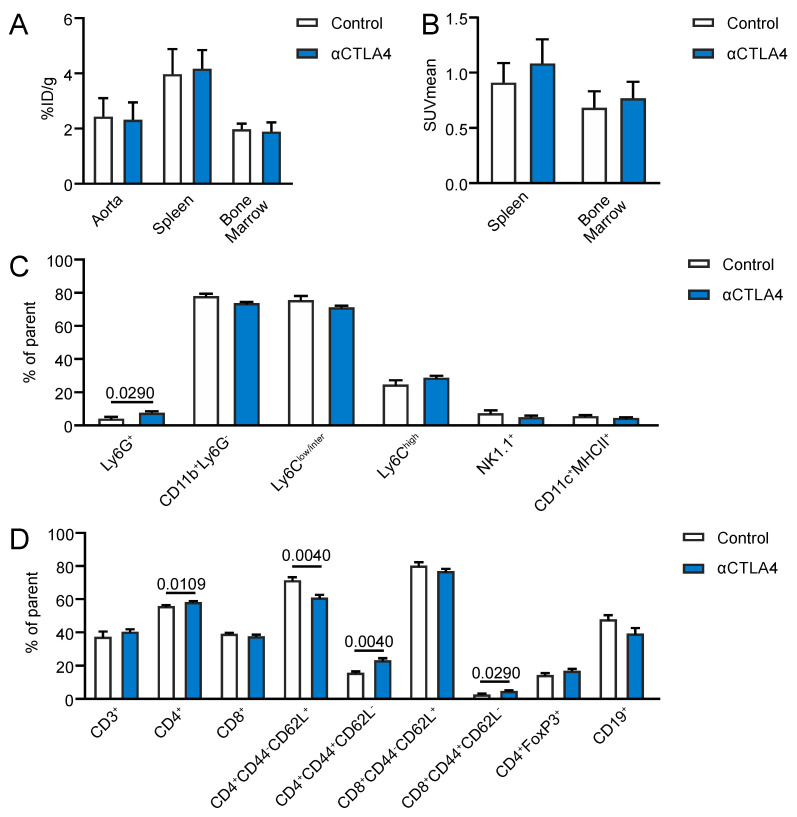
Antibody-mediated inhibition of CTLA4 induces an activated T cell profile in hyperlipidemic mice and does not affect monocyte/macrophage-driven inflammation. (**A**) Ex vivo gamma counting of the aorta, spleen, and bone marrow (*n* = 8). (**B**) Positron Emission Tomography—Computed Tomography revealed no differences in the ^18^F-fluorodeoxyglucose uptake in the spleen and bone marrow of αCTLA4-treated mice (*n* = 8). (**C**,**D**) Flow cytometry of cells from the spleen of control and αCTLA4-treated mice (*n* = 5–9).

To analyze whether the antibody-mediated inhibition of CTLA4 affected the monocyte/macrophage-driven vascular inflammation, ^18^F- fluorodeoxyglucose (FDG) PET/CT imaging was performed in *ApoE^−/−^* mice that were treated biweekly with anti-CTLA4 antibodies or PBS (control) for 4 weeks. No differences in the aortic ^18^F-FDG uptake were observed between the αCTLA4 and control mice (Figure 1A). The ^18^F-FDG uptake in the spleen and bone marrow was also unaffected by the antibody-mediated blockage of CTLA4 (Figure 1A,B). As ^18^F-FDG PET is widely used to analyze monocyte/macrophage-driven vascular and systemic inflammation in atherosclerosis, these data indicate that the antibody-mediated inhibition of CTLA4 did not affect the monocyte/macrophage-driven inflammation [16,17]. Flow cytometry of cells from the aorta demonstrated no differences in the vascular macrophage numbers, or the number of Ly6C^low/inter^ monocytes or Ly6C^high^ monocytes (Supplemental Figure S1a–c), which confirmed the ^18^F-FDG PET results. Together, these data show that αCTLA4 treatment did not affect the monocyte/macrophage-driven vascular or systemic inflammation in hyperlipidemic mice.

### 3.2. Antibody-Mediated Inhibition of CTLA4 Induces an Activated T Cell Profile in Hyperlipidemic Mice

To further assess the effects of the antibody-mediated blockage of CTLA4 on immune cell populations during hyperlipidemic conditions, flow cytometry was performed on the blood and lymphoid organs of αCTLA4-treated *Ldlr^−/−^* mice. In the spleen, a small but statistically significant increase in neutrophils (Ly6G^+^) was observed after the CTLA4 treatment (Figure 1C). Monocyte populations, natural killer (NK; NK1.1^+^) cells, and dendritic cells (DC; MHCII^+^CD11c^+^) were unaffected (Figure 1C). No significant differences in the total splenic CD3^+^ T cells were observed, but a minor increase in the CD4^+^ T cells was observed, whereas the CD8^+^ T cells and CD4^+^FoxP3^+^ regulatory T cells were not altered (Figure 1D). Phenotypical analyses of CD4^+^ and CD8^+^ T cells demonstrated a decrease in the naïve CD4^+^CD44^-^CD62L^+^ T cells, whereas CD4^+^CD44^+^CD62L^–^ and CD8^+^CD44^+^CD62L^–^ effector memory T cells increased (Figure 1D). Circulating effector memory CD8^+^ T cells and regulatory T cells were increased, whereas other T cell populations and B cells were unaffected (Supplemental Figure S1d). Together, these data demonstrate that anti-CTLA4 treatment induces an activated T cell profile in hyperlipidemic mice.

### 3.3. CTLA4 Inhibition Aggravates Endothelial Activation

**Figure 2:**
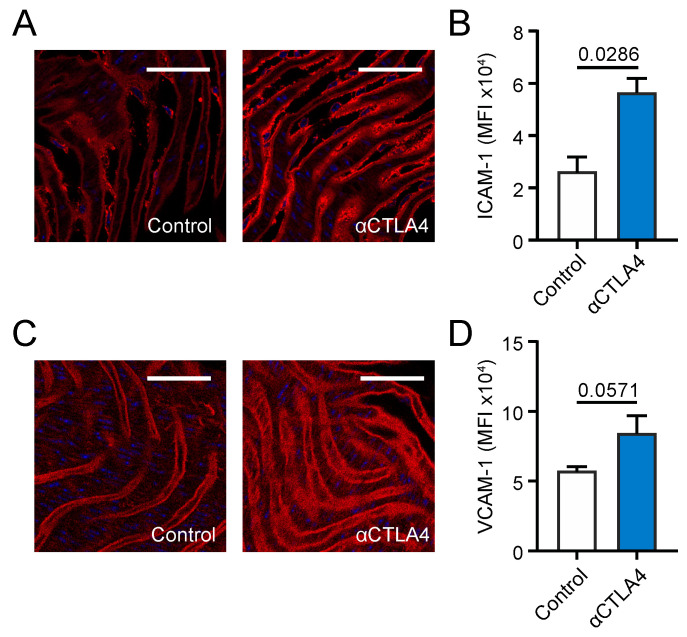
Antibody-mediated inhibition of CTLA4 aggravates endothelial activation in Ldlr^−/−^ mice. (**A**) Representative pictures of the en face expression of ICAM1 on the endothelium of the abdominal aorta. Scale bar: 50 µm. (**B**) Quantification of the en face expression of ICAM1 by confocal microscopy on the endothelium of the abdominal aorta (*n* = 4). (**C**) Representative pictures of the en face expression of VCAM1 on the endothelium of the abdominal aorta. Scale bar: 50 µm. (**D**) Quantification of the en face expression of VCAM1 by confocal microscopy on the endothelium of the abdominal aorta (*n* = 4).

Endothelial activation, which is characterized by the increased expression of adhesion molecules and the weakening of cell–cell junctional integrity, is one of the early steps in the development of atherosclerosis [15]. Therefore, the en face expression of vascular cell adhesion molecule (VCAM) 1 and intercellular adhesion molecule (ICAM) 1 on the aortic endothelium of αCTLA4-treated Ldlr^−/−^ mice was analyzed by confocal microscopy. The expression of ICAM1 increased 2.15-fold upon the antibody-mediated inhibition of CTLA4 (Figure 2A,B). A trend (*p* = 0.057) towards the increased expression of VCAM1 was observed in αCTLA4-treated Ldlr^−/−^ mice (Figure 2C,D), reflecting an activated endothelial phenotype. In addition, cell–cell junctions were analyzed by VE-cadherin staining. No major differences in VE-cadherin continuity were found (Supplemental Figure S2a,b). Thus, αCTLA4 treatment induced an activated endothelial phenotype in hyperlipidemic mice.

### 3.4. Antibody-Mediated Inhibition of CTLA4 Aggravates Atherosclerosis in the Aortic Arch

**Figure 3:**
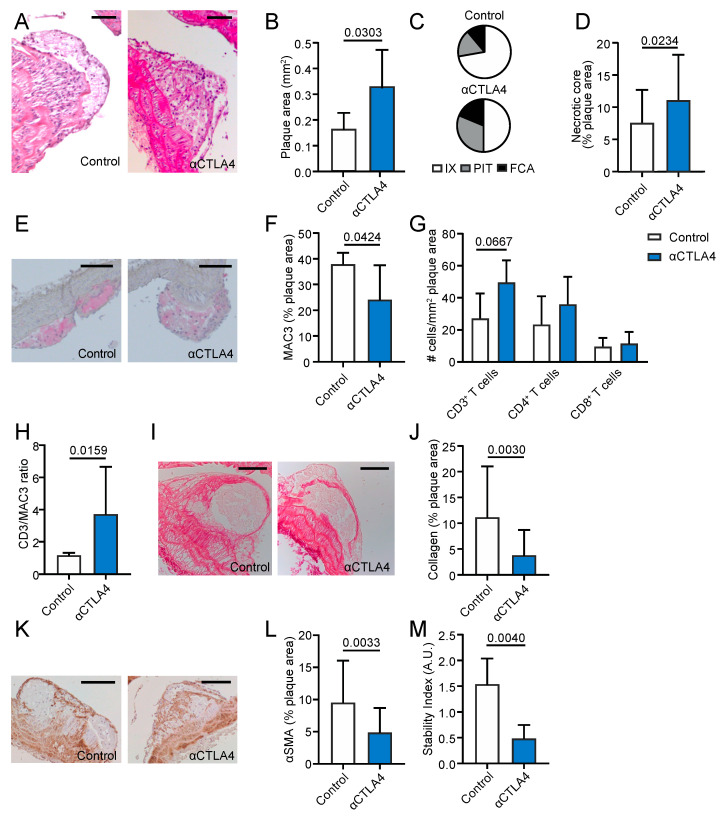
Antibody-mediated inhibition of CTLA4 aggravates initial atherosclerosis in the aortic arch. (**A**) Representative pictures of atherosclerosis in the brachiocephalic trunk (haematoxylin and eosin staining). Scale bar: 100 μm. (**B**) Atherosclerotic plaque area in the aortic arch (*n* = 5–9). (**C**) Morphological analysis of the atherosclerotic plaque phenotype according to the Virmani Classification. (**D**) Quantification of necrotic core area in plaques. (**E**) Representative pictures of the plaque macrophage content. Scale bar: 100 μm. (**F**) Quantification of plaque macrophage content. (**G**) Immunohistochemical quantification of CD3^+^, CD4^+^, and CD8^+^ cells. (**H**) The CD3/MAC3 ratio in the plaques. (**I**) Representative pictures of plaque collagen content. Scale bar: 100 μm. (**J**) Quantification of the collagen content of the atherosclerotic lesions. (**K**) Representative pictures of plaque smooth muscle cell (αSMA^+^) content. Scale bar: 100 μm. (**L**) Quantification of smooth muscle cell (αSMA^+^) content of the atherosclerotic lesions. (**M**) The stability index (smooth muscle actin positive area %/necrotic core area %). FCA = fibrous cap atheroma, IX = intimal xanthoma, PIT = pathological intimal thickening.

Atherosclerosis was analyzed in the aortic arch and its main branch points, which mostly contain early atherosclerotic lesions at the age of 18 weeks. The antibody-mediated blockage of CTLA4 induced a 2.0-fold increase in the atherosclerotic lesion area (Figure 3A,B). Morphological analysis of the plaque phenotype demonstrated a decrease in the intimal xanthomas (72.2% (control) vs. 50% (αCTLA4)), which is the most initial atherosclerotic lesion. Accordingly, an increase in more advanced lesions—e.g., pathological intimal thickenings (16.7% (control) vs. 31.3% (αCTLA4)) and fibrous cap atheromas (11.1% (control) vs. 16.7% (αCTLA4))—was observed, indicating that αCTLA4 treatment promoted the progression of atherosclerosis (Figure 3C). In accordance with the more advanced plaque phenotype, a 3.1-fold increase in the necrotic core area was observed upon the antibody-mediated inhibition of CTLA4 (Figure 3D). Plaque immune cell analysis revealed a significant decrease in the macrophage content (Figure 3E,F) and a trend towards increased CD3^+^ T cell numbers in αCTLA4-treated mice (*p* = 0.06) (Figure 3G). No differences in the CD4^+^ and CD8^+^ T cells and neutrophils were observed (Figure 3G, Supplemental Figure S2a). The CD3^+^/MAC3^+^ ratio increased after the αCTLA4 treatment (Figure 3H). To assess the plaque stability, the smooth muscle actin and collagen content were analyzed. A 48.5% decrease in the collagen content (Figure 3I,J) and a 56.1% reduction in the smooth muscle content (Figure 3K,L) were observed in mice that received CTLA4 blockage, which resulted in a decreased plaque stability index (smooth muscle actin positive area %/necrotic core area %) (Figure 3M). Proliferating (Ki67^+^) cells were slightly reduced after treatment, whereas apoptotic (TUNEL^+^) cells were unaffected (Supplementary Figure S3b,c). Together, these data demonstrate that the antibody-mediated inhibition of CTLA4 increases the atherosclerotic lesion area and promotes the progression of early atherosclerotic plaques towards an advanced and more unstable plaque phenotype.

### 3.5. Inhibition of CTLA4 Aggravates Plaque Inflammation and Progression in the Aortic Root

**Figure 4:**
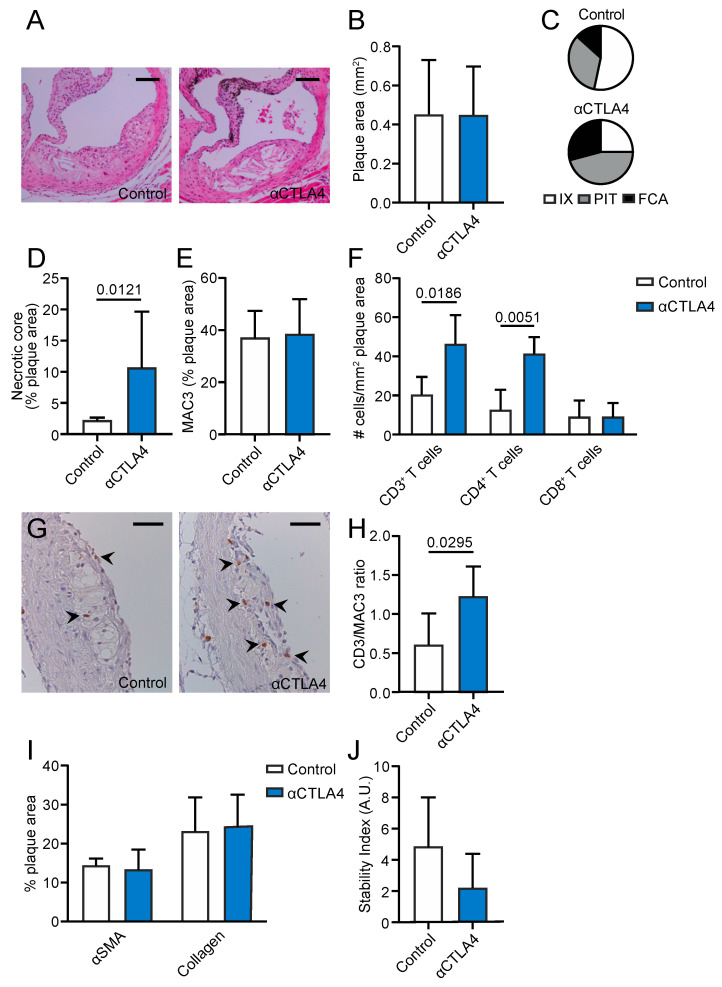
Antibody-mediated inhibition of CTLA4 promotes the progression of atherosclerosis in the aortic root. (**A**) Representative pictures of plaques in the aortic root (haematoxylin and eosin staining). Scale bar: 200 μm. (**B**) Atherosclerotic plaque area in the aortic root (*n* = 5–9). (**C**) Morphological analysis of the atherosclerotic plaque phenotype according to the Virmani Classification. (**D**) Quantification of necrotic core area in plaques. (**E**) Quantification of plaque macrophage content. (**F**) Immunohistochemical quantification of CD3^+^, CD4^+^, and CD8^+^ cells. (**G**) Representative pictures of CD3^+^ cells in the plaque. Scale bar: 150μm. (**H**) The CD3/MAC3 ratio in the plaques. (**I**) Quantification of smooth muscle actin and collagen content in the plaques. (**J**) The stability index (smooth muscle actin positive area %/necrotic core area %). FCA = fibrous cap atheroma, IX = intimal xanthoma, PIT = pathological intimal thickening.

In the aortic root, where more established lesions are present after 12 weeks of diet, no differences in plaque size were observed (Figure 4A,B). The morphological classification of the plaque phenotype demonstrated that the incidence of intimal xanthomas was decreased (54% (control) vs. 25% (αCTLA4)) in anti-CTLA4-treated mice, whereas pathological intimal thickenings (33% (control) vs. 46% (αCTLA4)) and fibrous cap atheromas (13% (control) vs. 29% (αCTLA4)) were increased (Figure 4C). Corresponding with the more advanced plaque phenotype, a 4.75-fold increase in the necrotic core area was observed upon CTLA4 blockage (Figure 4D). However, no differences in the macrophage content were found (Figure 4E). The plaque CD3^+^ T cell content significantly increased, especially due to the increased CD4^+^ T cell numbers (Figure 4F). CD8^+^ T cells were unaffected (Figure 4F,G). This increase in CD3^+^ T cells was also reflected by an increased CD3^+^/MAC3^+^ ratio after the antibody-mediated inhibition of CTLA4 (Figure 4H), indicating a shift towards lymphoid-driven inflammation in the plaque. No changes in Ly6G^+^, Ki67^+^, or TUNEL^+^ cells were found (Supplemental Figure S4a–c). Overall, the smooth muscle actin, collagen content, and stability index did not change after treatment (Figure 4I,J). However, when separating the initial and advanced plaques, differences could be observed in the αSMA, which is lower in advanced plaques of the treated group compared to the control, and macrophage content, which decreases as the plaques progresses in the treated group, but remains unchanged in the control mice (Supplementary Figure S4d,e). These data indicate that the antibody-mediated inhibition of CTLA4 also aggravates plaque inflammation and progression in more advanced atherosclerosis.

## 4. Discussion

Here, we report that the short-term antibody-mediated inhibition of CTLA4 aggravates experimental atherosclerosis by accelerating the progression of initial plaques towards more advanced and unstable lesions. The inhibition of CTLA4 did not affect the monocyte/macrophage-driven inflammation but induced an activated T cell profile and increased the CD3^+^/MAC3^+^ ratio in plaques, reflecting a strong T cell-driven inflammation.

Several factors may have contributed to the aggravation of atherosclerosis upon the antibody-mediated inhibition of CTLA4. First, we observed that the blockage of CTLA4 increased the expression of the adhesion molecule ICAM1 on the aortic endothelium of hyperlipidemic mice. Endothelial activation, characterized by an increased expression of adhesion molecules, is one of the earliest steps in the development of atherosclerotic lesions and facilitates the recruitment of immune cells to sites of vascular inflammation, which enhances plaque formation [18,19,20]. Taggart et al. previously reported that the antibody-mediated inhibition of immune checkpoint proteins increased the expression of adhesion molecules on the tumor endothelium via T-cell-derived IFNγ-driven mechanisms, which enhanced immune cell recruitment to the tumor [21]. Our data suggest that similar mechanisms enhanced the expression of adhesion molecules on the endothelium of the large arteries, thereby promoting leukocyte migration into the arterial wall. In accordance with our findings, previous studies showed that the pharmacological inhibition of CD80/86-CD28 interactions by the CTLA4-Ig protein abatacept dose-dependently reduced the expression of ICAM1 in activated endothelial cells [22]. Abatacept also reduced the expression of this adhesion molecule in the aortic lysates of ApoE^−/−^ mice that were subjected to hyperhomocysteinaemia-accelerated atherosclerosis by limiting the expression of inflammatory cytokines [23]. In addition, the antibody-mediated blockage of CTLA4 increases the T cell motility in ICAM1-coated surfaces in vitro, suggesting that CTLA4 interaction normally inhibits T cell motility to limit T cell-APC interaction and thus reduce the T cell activation [24]. Lastly, He et al. found that the blockage of CTLA4 impaired the regulatory T cell-mediated suppression of endothelial activation, thereby increasing the expression of inflammatory cytokines and adhesion molecules [25]. Thus, in initial atherosclerosis, anti-CTLA4 treatment may indirectly increase the endothelial adhesion molecule expression by aggravating systemic and vascular T cell-driven inflammatory responses.

The antibody-mediated inhibition of CTLA4 also induced an activated T cell profile in hyperlipidemic mice, without affecting the monocyte/macrophage-driven inflammation, which confirmed the well-known inhibitory function of this immune checkpoint protein in the regulation of T cell activation. CTLA4 blockage increased effector memory T cells in our study, which is similar to the observations in cancer patients who receive the CTLA4 inhibitor ipilimumab [26,27]. In humans, circulating CD4^+^ effector memory T cell numbers correlate to the intima-media thickness and the occurrence of myocardial infarction and the number of CD4^+^ T cells in the plaque increases during lesion progression [28,29]. Additionally, in both ApoE^−/−^ and Ldlr^−/−^ mice, circulating CD4^+^ effector memory T cells correlated with the atherosclerotic lesion area, implicating that the increased abundance of T cells that we found in anti-CTLA4-treated mice contributed to the accelerated progression of atherosclerosis in these mice [28]. Our observations are in line with previous studies that explored the role of the CD80/86-CD28 and -CTLA4 pathways in atherosclerosis. For example, the T cell-specific overexpression of CTLA4 reduced atherosclerosis in ApoE^−/−^ mice and limited the numbers of CD4^+^ T cells and macrophages in the plaque and decreased systemic T cell activation [30]. Similarly, abatacept improved hyperhomocysteinemia-accelerated atherosclerosis in ApoE^−/−^ mice by limiting the T-cell driven inflammatory responses [23]. Abatacept also reduced the atherosclerotic burden in ApoE3*Leiden mice and induced a clinically favorable plaque phenotype that is low in inflammatory cells and high in smooth muscle cell content [13]. In a different model, the antibody-mediated inhibition of CTLA4 aggravated the post-interventional lesion formation in the femoral artery of ApoE3*Leiden mice by increasing the T cell-driven inflammation [13]. In our study, mice were subjected to short-term αCTLA4 treatment, and in the aortic root no differences in the macrophage, αSMA, and collagen content were observed. However, phenotypic analysis showed that the treated mice had less αSMA content in more advanced plaques. This was accompanied by a decrease in the macrophage content in the advanced lesions of treated mice, whereas the percentage did not differ in the control group. Increased macrophage death is one of the hallmarks of plaque progression, and could explain the decrease in macrophage content, the increase in TUNEL^+^ cells, and the shift towards a more T-cell driven inflammation, all of which are features of plaque progression. Together, these studies and our findings identify a protective role for CTLA4 in atherosclerosis, where it potentially decreases lymphoid-driven inflammation, and thereby limits macrophage death and plaque progression. Together, these data suggest that agonizing the function of this co-inhibitory molecule is a promising therapeutic strategy to temper the inflammatory response that drives atherosclerosis.

Nowadays, immune checkpoint inhibitors are considered the standard-of-care for several cancers, including melanoma and non-small cell lung cancer [10,11]. Immune checkpoint inhibitors are monoclonal antibodies directed against co-inhibitory molecules, in particular CTLA4, programmed cell death (PD) 1, and PD ligand (PDL) 1. By inhibiting the natural brake on T cell activation, these antibodies induce potent anti-tumor immune responses, which has led to unprecedented response rates in cancer patients [10,11]. In our study, we found that the antibody-mediated blockage of CTLA4 induced an activated T cell profile in hyperlipidemic mice, which is similar to the observations in cancer patients who receive the αCTLA4 antibody ipilimumab [26,27]. Whether patients who are treated with ipilimumab have an increased risk of developing atherosclerotic cardiovascular disease is currently unknown, as atherosclerosis develops gradually over years or decades and immune checkpoint inhibitors have been implemented in the clinic only in the past decade [31]. Moreover, the elderly, who often have subclinical atherosclerosis, and patients with a history of cardiovascular disease were excluded from most of the clinical trials that investigated ipilimumab in cancer patients [32]. Nevertheless, a meta-analysis of 22 trials that investigated the efficacy of another immune checkpoint inhibitor targeting the co-inhibitory PD1-PDL1 dyad in patients with lung cancer demonstrated that atherosclerotic cardiovascular disease occurred in 3% of the patients [33]. In addition, approximately 1% of the patients with lung cancer who were treated with anti-PD(L)1 agents developed a myocardial infarction or stroke within the first 6 months after the initiation of therapy, suggesting that these adverse events resulted from effects on existing atherosclerotic plaques and not from the de novo development of atherosclerotic lesions [34]. A recent autopsy study in 11 cancer patients who died from non-cardiovascular causes provided further insight into the pathophysiology of ICI-related atherosclerotic disease [35]. Anti-PD(L)1 or combined anti-PD(L)1 and anti-CTLA4 therapy increased the T cell/macrophage ratio in coronary atherosclerotic plaques, which reflects a strong T cell-driven inflammation that is associated with plaque instability [35,36]. Whether ICI therapy targeting CTLA4 induces similar changes in plaque inflammation in cancer patients is currently unknown and should be investigated in future clinical studies, as our study demonstrates that the inhibition of CTLA4 accelerates the progression of experimental atherosclerosis.

## Supplementary Materials

The following are available online at https://www.mdpi.com/2073-4409/9/9/1987/s1. Figure S1: Antibody-mediated inhibition of CTLA4 induces an activated T cell profile in hyperlipidemic mice and does not affect monocyte/macrophage-driven inflammation; Figure S2: Representative pictures of the *en face* expression of VE-cadherin on the endothelium of the abdominal aorta; Figure S3: Additional immunohistochemical quantifications of the aortic arch; Figure S4: Additional immunohistochemical quantifications of the aortic root.
