# Supplementary material for: Antibody-Mediated Inhibition of CTLA4 Aggravates Atherosclerotic Plaque Inflammation and Progression in Hyperlipidemic Mice
Source: Cells. 2020 Aug 29;9(9):1987. doi: 10.3390/cells9091987 (PMC7565685; doi:10.3390/cells9091987)
Supplement: Supplementary file 1 [file cells-09-01987-s001.pdf]

Figure S1

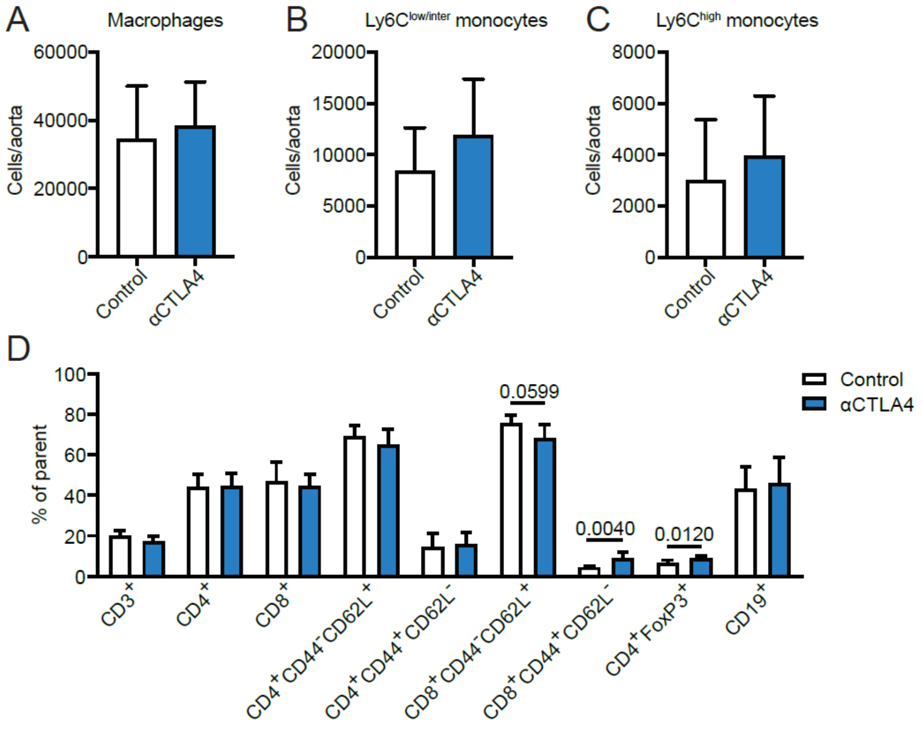

Figure S1. Antibody-mediated inhibition of CTLA4 induces an activated T cell profile in hyperlipidemic mice and does not affect monocyte/macrophage-driven inflammation. **(A, B, C)** Flow cytometry analysis of macrophages and monocytes in the aorta, after αCTLA4 treatment. There is no difference between the groups. **(D)** In the circulation, flow cytometry analysis indicated an increase in $CD8^+CD44^+CD62L^-$ effector memory cells and an increase in $CD4^+FoxP3^+$ regulatory T cells after αCTLA4 treatment. Other T cell populations and B cells were not affected.

Figure S2:

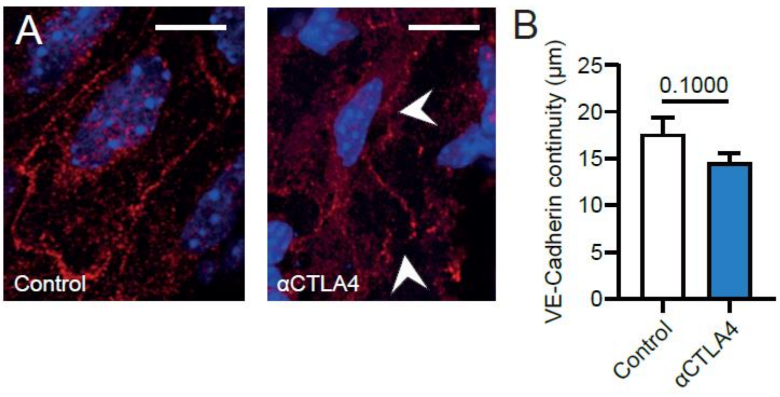

Figure S2. (A) Representative pictures of the en face expression of VE-cadherin on the endothelium of the abdominal aorta. Arrow heads indicate disrupted cell-cell junctions. Scale bar: 10 µm. B) Quantification of VE-cadherin continuity by confocal microscopy on the endothelium of the abdominal aorta ($n = 3$).

Figure S3

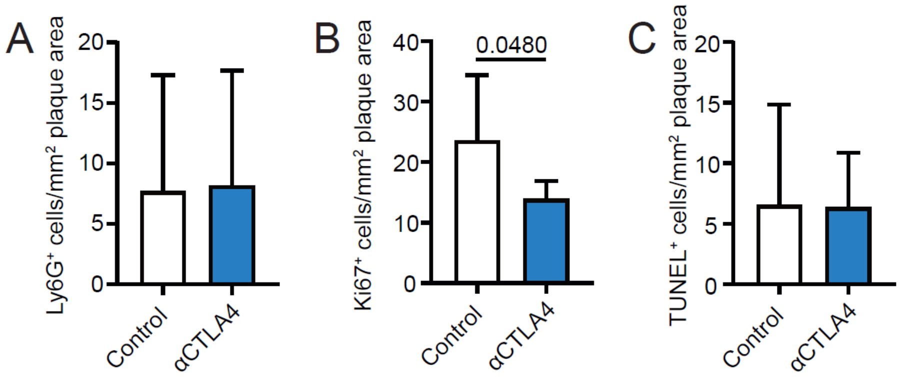

Figure S3. Additional immunohistochemical quantifications of the aortic arch. (**A**) Quantification of Ly6G+ neutrophils in the plaque. (**B**) Quantification of Ki67+ proliferating cells in the plaque. (**C**) Quantification of TUNEL+ apoptotic cells.

Figure S4:

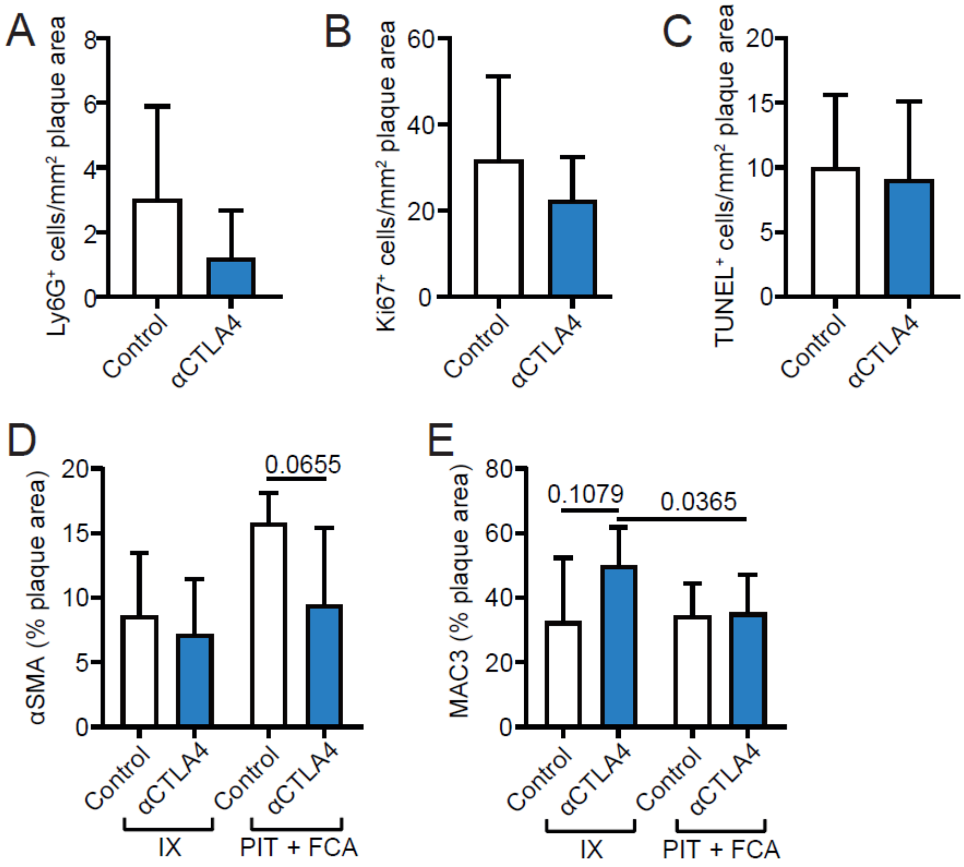

Figure 4. Additional immunohistochemical quantifications of the aortic root. (**A**) Quantification of Ly6G+ neutrophils in the plaque. (**B**) Quantification of Ki67+ proliferating cells in the plaque. (**C**) Quantification of TUNEL+ apoptotic cells. (**D**) Quantification of αSMA per plaque phenotype. (**E**) Quantification of MAC3 per plaque phenotype.
